# Formation of Hierarchical Nanoporous Gold via Selective Dissolution and Dealloying of Ternary (Au–Ag)–Ge Two-Phase Hypereutectic Alloy

**DOI:** 10.1021/acs.cgd.5c00382

**Published:** 2025-06-11

**Authors:** Lotan Portal, Iryna Polishchuk, Rotem Zilberberg, Maria Koifman-Khristosov, Alexander Katsman, Boaz Pokroy

**Affiliations:** † Department of Materials Science and Engineering, 108411 Technion − Israel Institute of Technology, Haifa 32000, Israel; ‡ Russell Berrie Nanotechnology Institute, Technion − Israel Institute of Technology, Haifa 32000, Israel; § The Nancy and Stephen Grand Technion Energy Program, Technion − Israel Institute of Technology, Haifa 3200003, Israel

## Abstract

Nanoporous (np) metal foams exhibit unique three-dimensional bicontinuous structures characterized by nanometric pores and ligaments, remarkable surface area, catalytic activity, and mechanical robustness. Traditionally, np foams are fabricated by selective dissolution of less-noble elements from metal alloys, leaving behind noble metal frameworks. This study pioneers the use of a ternary Au–Ag–Ge eutectic alloy to synthesize hierarchically complex np-Au crystals. Through a two-step selective dissolution technique involving the removal of Ge followed by dealloying Ag, we achieved a dual-level porosity with controlled morphology. The resulting microstructures feature pore and ligament sizes spanning 10–50 nm to 100–350 nm. By varying the dissolution methods and durations, pore sizes were further refined to approximately 20 nm. High-resolution XRD and EDX confirm the dissolution of most of the Ge and Ag from the alloy, while cross-sectional SEM imaging reveals the complexity of the hierarchical architecture. The hierarchical np-Au crystals demonstrated a dramatic enhancement in catalytic activity, up to 10-fold compared to binary Au–Ge-derived np-Au and 2-fold relative to conventional np-Au. This advancement underscores the potential of ternary eutectic alloys for creating multifunctional nanostructures and offers a promising avenue for tuning the catalytic performance across various applications.

## Graphical abstract

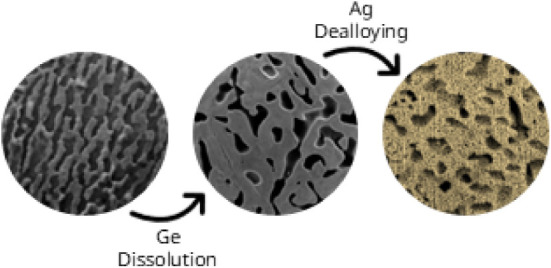

## Introduction

Nanoporous (np) metal foams^1^ are widely used across a variety of scientific and engineering fields, including catalysis,^2–8^ energy storage,^2,9,10^ sensors,^2,11–13^ actuators,^14–16^ and sound and energy absorption.^3,17^ These three-dimensional (3D) bicontinuous structures provide a large surface area, high stiffness-to-volume ratios, and enhanced thermal and electrical conductivity.^17^ Their formation has been extensively explored in various metals, such as gold (Au), platinum (Pt), silver (Ag), and nickel (Ni). Although numerous synthesis methods exist today,^18^ the most conventional approach to synthesize metal foams remains the top-down selective dealloying, where at least one less noble metal is selectively removed from a homogeneous metallic alloy via electrochemical dissolution or selective corrosion in acidic or basic solutions. The rapid passivation of low-coordinated atoms of the less noble element leads to the detachment of the atoms into the dealloying solution, creating vacancies in the previously homogeneous alloy. This process facilitates the rapid diffusion and reorganization of noble metal atoms, forming nanoscale ligaments. The final morphology of the metallic foam is shaped by the velocity of the dealloying front of the dissolved less noble metal and the diffusion rate of the remaining noble atoms. Consequently, structural characteristics are influenced by factors such as the applied electrochemical voltage during electrochemical dissolution, the type and concentration of the dealloying solution during chemical dealloying, as well as the initial alloy composition and temperature.^19^ Additionally, hierarchical porosity can be achieved through variations in synthesis conditions,^18,20,21^ further refining the foam’s functional properties.^22,23^

Nanoporous Au (np-Au) catalysts are widely used in various chemical reactions due to their exceptional catalytic properties. Np-Au demonstrates high catalytic activity in oxidation reactions, such as low-temperature CO^24,25^ or methanol^4,26^ oxidation, as well as in the reduction of aromatic nitro compounds.^27,28^ Its biocompatibility, nontoxicity,^29,30^ and status as a low-cost, reusable, and green catalyst make Au an attractive choice for numerous chemical reactions. Among the various methods for synthesizing np-Au, the most common involves the dealloying of Ag from Au–Ag alloys using high-concentration nitric acid.^19,31,32^ This top-down approach yields a 3D bicontinuous porous morphology with ligament sizes ranging from 30 to 50 nm, depending on the alloy composition and nitric acid concentration. Several attempts have been made to utilize ternary and multicomponent alloys for the preparation of nanoporous metal structures. Among these are Ag–Au–Pt ternary alloys with a small percentage of platinum,^33,34^ which, after electrochemical dealloying, yielded an extremely high surface area nanoporous metal with pore sizes smaller than 4 nm. Another example is ternary metallic glass ribbons, such as Au_55_Cu_25_Si_20_,^8,35^ which were dealloyed through the selective dissolution of less noble Cu. This process transformed the ribbons into metastable gold silicide. Cone-like porous protrusions of gold silicide formed on the surface of the amorphous metallic ribbons, which subsequently decomposed into gold nanoparticles and amorphous SiO_2_, together forming 3D nanoporous structures.

The advantage of the work by Hu et al.^8,35^ lies in their use of a relatively low concentration of less noble metal (only 25% Cu), compared to the usual percolation threshold of approximately 55% Ag in Au–Ag alloys required for the creation of bicontinuous nanoporous gold. However, in all of these cases, the traditional one-step selective dealloying (chemical or electrochemical) of the less noble element was employed.

A unique approach to synthesizing np-Au crystals was pioneered by Khristosov et al.,^36,37^ in which the authors employed the selective dissolution of Ge from Au–Ge eutectic micron-size particles in basic solutions at room temperature. The selective removal of the Ge phase from the Au–Ge eutectic microstructure produces cylindrical, closed pores and Au ligaments with sizes ranging from 50 to 350 nm. As shown in our recent work,^38^ the pore and ligament sizes of the np-Au droplet-like crystals synthesized by this unique method can be fine-tuned by changing the undercooling of the eutectic melt during solidification.^39^ We also demonstrated that enhancing the catalytic activity of these crystals is possible by reducing the pore and ligament sizes, which can be achieved by increasing the undercooling of the eutectic melt. The undercooling can be adjusted in various ways, such as increasing the cooling rate, decreasing the layer thickness and material’s volume, or selecting an alloy composition closer to the eutectic point.

In this work, we aim to further improve the catalytic activity of np-Au crystals synthesized from eutectic microstructures by creating hierarchical porous morphologies. The hierarchical nanoporous gold (np-Au) was first synthesized by Ding and Erlebacher,^40^ who developed a bimodal pore size distribution through a sequential dealloying approach. This method involved several stages: first, the chemical dealloying of silver to create large-pore-size np-Au, followed by thermal coarsening. Silver plating was then used to fill the pores, and subsequent heat treatment realloyed the gold and silver. Finally, a second dealloying step dissolved the plated silver, resulting in a hierarchical nanoporous structure.

An alternative fabrication method, stepwise dealloying, was reported by Qi and Weissmüller.^20^ Their approach began with incomplete dealloying of a dilute Au–Ag alloy, which retained a significant Ag fraction within a nanoporous Ag–Au alloy. Thermal coarsening was applied to generate larger pores, and in the second dealloying step, a higher potential removed the remaining Ag, producing a secondary hierarchy of nanopores within each larger ligament.

In the present work, we introduce a more streamlined process for synthesizing hierarchical np-Au, reducing the number of intermediate stages while achieving comparable structural complexity.

To achieve this, we utilized the ternary (Au–Ag)-Ge alloy as a two-phase eutectic system that can be dealloyed through separate processes. We combine the conventional Au–Ag dealloying method with the selective dissolution of Ge from (Au–Ag)–Ge eutectic microstructures to synthesize np-Au crystals. This work reports the formation of hierarchical np-Au morphology with a dual-level porous structure: the first level features pore and ligament sizes ranging from 150 to 300 nm, while the second level exhibits porosity in the range of 20–35 nm. Additionally, we demonstrate that hierarchical np-Au morphology can be achieved through either a one- or two-step dissolution process. While both methods result in the formation of hierarchical np-Au crystals with intricate 3D bicontinuous structures, the two-step dissolution approach provides greater control over pore and ligament dimensions by fine-tuning the relationship between dealloying duration and ligament coarsening. Catalysts synthesized via the two-step dissolution method exhibit enhanced catalytic performance, further advancing the potential applications of np-Au.

## Experimental Section

### Sample Preparation

The synthesis of hierarchical round nanoporous Au (np-Au) crystals was carried out using the following method: thin layers of Au–Ag (50:50 at. %, 99.999% pure, ACI Alloys) and Ge (99.999% pure, Sigma-Aldrich) were evaporated on a (001) Si substrate with a thick SiO_2_ diffusion barrier layer (100 nm) to prevent interactions between the thin layers and the substrate. The evaporation process was performed using a PVD-4 thermal evaporator (VINCI Technologies) at room temperature under a vacuum of 5 × 10^–6^ bar, with the chamber heating up to a maximum of 185 °C during the process. Standard layer thicknesses were 200 nm for the Au–Ag layer and 75 nm for the Ge layer, yielding a hypereutectic composition of approximately 28.7 wt % Ge (while the eutectic composition is 15.6 wt % Ge).^41^

To enable full liquidation and dewetting of the thin layers, thermal annealing was performed using a MILA-5000 ULVAC-RIKO rapid thermal annealer (RTA) at 750–850 °C for 5 min under low vacuum (1.5–3 mbar) with a constant gas flow of forming gas (FG, 5%H_2_ + 95%Ar, 99.99%, Maxima). The samples were heated at a rate of 10 °C s^–1^ and cooled at an average rate of 0.7 °C s^–1^, resulting in the solidification of hypoeutectic round crystals.

Hierarchical np-Au morphology was achieved by the selective dissolution of Ge and Ag at room temperature using two different methods. The first method, termed “two-step dissolution”, involved the selective dissolution of Ge by immersion in a basic piranha solution of NH_4_OH (AR, Bio-Lab Ltd.) and H_2_O_2_ (30% w/v, Panreac) at 1:25% vol ratio for 2 h, followed by dealloying of Ag from the remaining Au–Ag ligaments using HNO_3_ (70%, Bio-Lab Ltd.). The second method, termed ″one-step dissolution”, combined the dissolution of both Ge and Ag by immersion in HNO_3_ directly, bypassing the initial step in the basic solution.

### Sample Characterization

The morphology of the round nanoporous crystals was investigated using a Zeiss Ultra-Plus field emission gun high-resolution scanning electron microscope (HR-SEM, WD = 3.8–4 mm, 4 keV). Average pore sizes were measured from HR-SEM micrographs using ImageJ for image analysis. A minimum of 200 representative pores were measured for each sample. As-evaporated layer thicknesses were measured from cross-sectional lamellae using FEI Tecnai G2 T20 S-Twin high-resolution transmission electron microscopy (HR-TEM, 200 keV) in scanning transmission electron microscopy mode (STEM). Lamellae preparation and cross-sectional imaging of full crystals were performed using a Thermo Fisher Helios 5 Plasma Focused Ion Beam (PFIB). Crystallographic analysis of samples before and after dissolution was conducted using high-resolution X-ray diffraction (HR-XRD) on a Rigaku SmartLab 9 kW, employing monochromatic radiation at 1.5406 Å. The chemical composition of the different elements at various dissolution durations was analyzed using energy-dispersive X-ray spectroscopy (EDX, X-Max 80, Oxford Instruments, accelerating voltage of 20 kV, and working distance of 8.5 mm) mounted on an HR-SEM. Further study of the chemical composition of the crystals’ cross sections was conducted using an Oxford Ultim-MAX electron-dispersive X-ray spectroscope (EDX), mounted on a PFIB. The Ag concentration in the acidic dissolution solutions was measured using inductively coupled plasma optical emission spectroscopy (ICP-OES, Thermo Scientific). The HNO_3_ etching solution was diluted to achieve <3% in DI water. The catalytic activity of the selected samples was assessed through weight loss measurements. A beaker containing 10 mL of H_2_O_2_ solution was weighed before and after the addition of the nanoporous gold (np-Au) catalyst. Weight measurements were taken hourly to monitor the reduction in the weight of the H_2_O_2_ solution due to oxygen release. Two controls were included in the experiments to evaluate the influence of external factors on the decomposition reaction: (1) A Si/SiO_2_ substrate was measured to determine the substrate’s contribution to the reaction in the absence of any Au catalyst. (2) A beaker without any catalyst served as a baseline for the natural decomposition of H_2_O_2_ at room temperature when exposed to air.

## Results and Discussion

The ternary Au–Ag–Ge system, as described by Hassam et al.,^41^ can be used to form a two-phase eutectic microstructure composed of lamellae of Au–Ag solid solution and inclusions of the Ge phase. To synthesize hierarchical np-Au crystals, thin layers of Au–Ag and Ge were evaporated to achieve a hypereutectic composition of 28.7 wt % Ge,^41,42^ followed by thermal annealing to induce full liquidation and dewetting of hypereutectic droplets on a Si/SiO_2_ substrate (for full synthesis description, see Experimental Section). The dewetting step resulted in droplet-like liquid alloys being distributed on the surface of the Si/SiO_2_ substrate. Rapid cooling of the hypereutectic melts at an average rate of 0.7 °C s^–1^ led to the immediate solidification of these droplets into round droplet-like crystals, as described thoroughly in the works of Khristosov et al.^36,37,43^ The final microstructures of these crystals consisted of primary Ge crystals and eutectics consisting of Au–Ag solid solution lamellae and cylindrical and globular Ge inclusions, as shown in Figure S3a. This structure is well manifested after the removal of Ge and the subsequent removal of Ag (Figures 3e,f and S3b,c). To create the hierarchical np-Au morphology, Ge and Ag were selectively removed from the system through two selective dissolution steps. First, the Ge was removed using a basic Piranha solution, as described above, resulting in the first level of porosity, as presented in Figure S3b. Second, Ag was dealloyed from the remaining Au–Ag lamellae by using 70% HNO_3_, forming the second level of porosity, as displayed in Figure S3c. Each dissolution step involved immersing the samples for 2 h. A similar sample with hypereutectic composition was synthesized using thin layers of Au and Ge, without Ag, where Au replaced the entire Au–Ag layer. This sample represents the binary two-phase system previously used to form np-Au morphology with a single level of porosity.^36–38^ The binary Au–Ge sample underwent the same thermal annealing and dissolution treatment as the ternary samples. Although immersion in acid was not required due to the absence of Ag, the dissolution step was still carried out to ensure consistent experimental conditions. Cross-sectional lamellae of the as-evaporated samples were prepared using a PFIB (see Figure S2). The thicknesses of the layers on the wafer were measured at several points to calculate the compositions of the final samples.

The final np-Au crystals derived from both binary Au–Ge and ternary Au–Ag–Ge systems were imaged by using high-resolution scanning electron microscopy (HR-SEM), as shown in Figure 1. The np-Au samples from the binary Au–Ge system exhibit a single level of porosity with large cylindrical pores ranging from 100 to 350 nm (Figure 1a–c). In contrast, the np-Au samples from the ternary Au–Ag–Ge system display two distinct levels of porosity (Figure 1d–f). The first level consists of large cylindrical pores with an average size similar to those in the binary Au–Ge crystals, formed by the selective dissolution of Ge. The second level features smaller pores, ranging from 15 to 50 nm, introduced during the second dissolution step, where Ag was dealloyed from the remaining Au–Ag ligaments using HNO_3_. Cross-sectional imaging of the internal morphology for both samples was obtained using a focused ion beam (FIB) coupled with HR-SEM. As seen in Figure 1c, the porous morphology within the binary system’s droplet remains consistent with the surface morphology. Similarly, Figure 1f reveals that the hierarchical morphology in the ternary alloy sample is preserved throughout the entire volume of the droplet.

**Figure 1:**
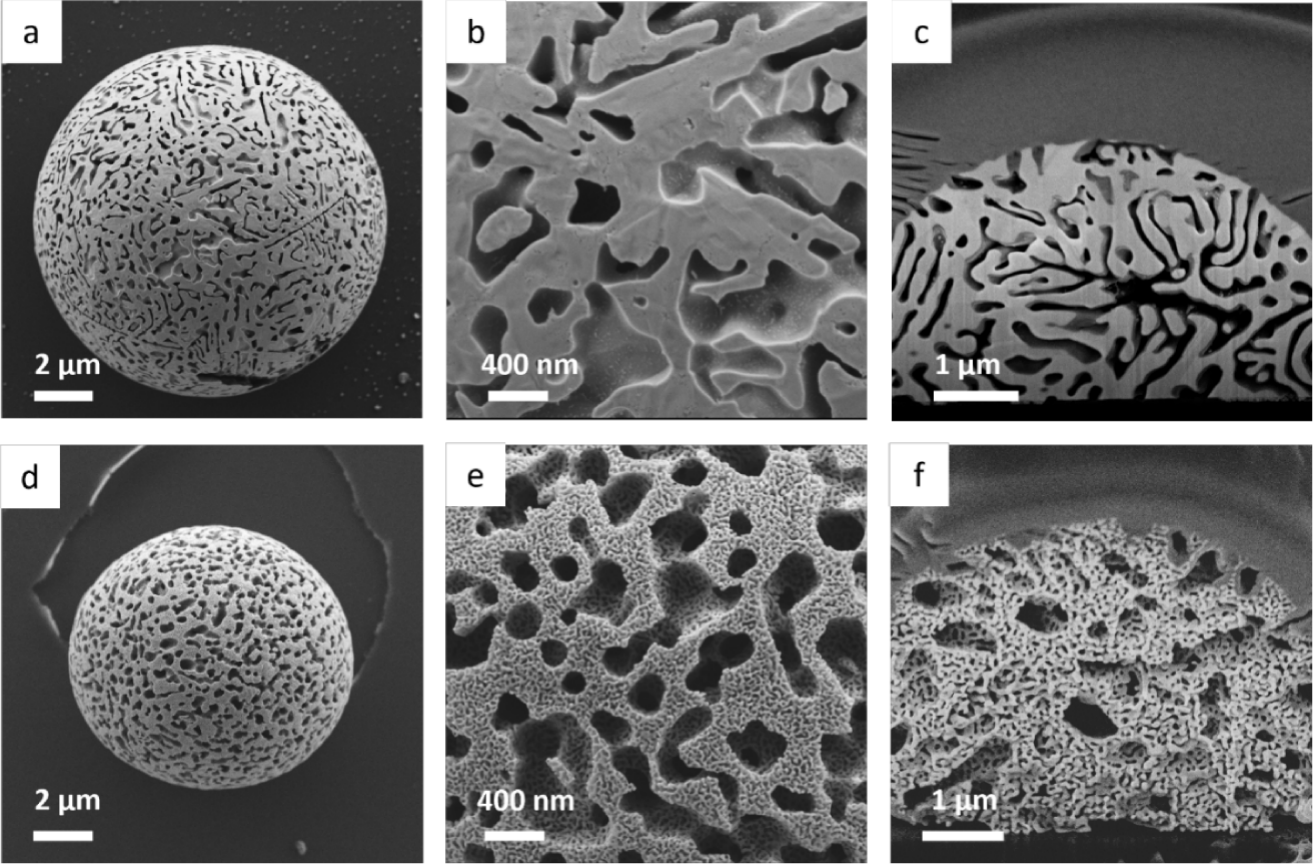
HR-SEM micrographs of np-Au morphologies: (a, b) surface morphology and (c) cross-section of np-Au crystal formed from the binary Au–Ge hypereutectic melt, showing one level of pores after selective dissolution of Ge; (d, e) surface morphology and (f) cross-section of np-Au droplet formed from the ternary (Au–Ag)–Ge hypereutectic melt, showing hierarchical porous morphology.

We further aimed to study the influence of different approaches to creating hierarchical np-Au crystals on their resulting morphology. To this end, a hypereutectic sample of (Au–Ag)-Ge was synthesized as described in the Experimental Section, involving annealing at 750 °C under a constant flow of FG (Ar), followed by rapid cooling to solidify the structure. The sample was then cut into two similar pieces, each subjected to a different approach of Ge and Ag dissolution to create a hierarchical morphology. In the first case, referred to as “two-step dissolution”, the selective dissolution of Ge was performed first using a basic solution, followed by the selective dealloying of Ag in the second step using HNO_3_. In the second case, referred to as “one-step dissolution”, both Ge and Ag were dissolved simultaneously in HNO_3_. In the standard procedure for both approaches, each sample was immersed in the corresponding dissolution solution for 2 h. Both dissolution approaches are described in detail in the Experimental Section. Additionally, an np-Au sample was synthesized using a binary Au–Ge alloy by decomposition of Ge in HNO_3_. This sample served as a reference for the hierarchical ternary samples. The final morphology in the binary Au–Ge case was a non-hierarchical porous structure characterized by a single scale of pores with an average size of 197 ± 87 nm (Figure 2a).

**Figure 2:**
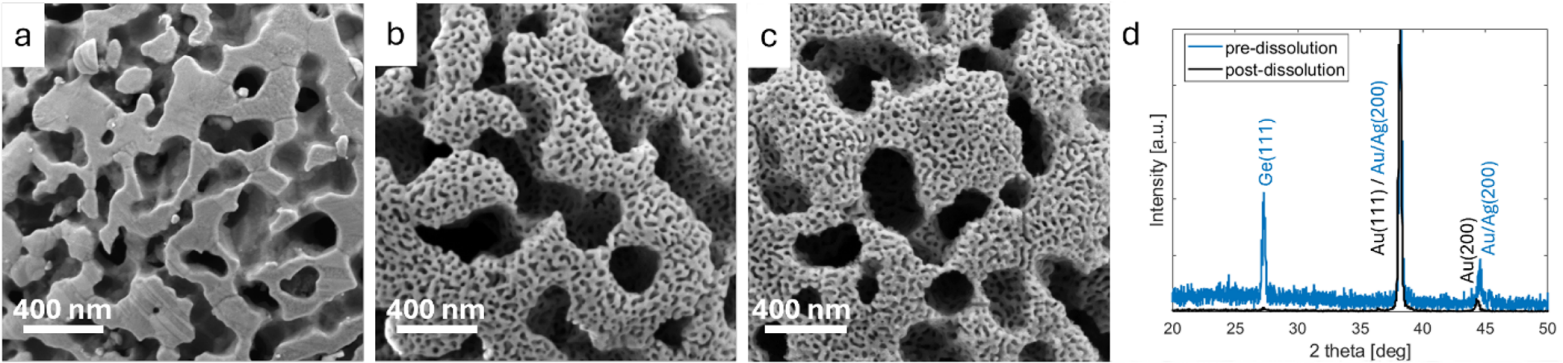
HR-SEM micrographs and XRD of np-Au crystals. (a) Binary Au–Ge sample synthesized by selective dissolution of Ge using HNO_3_ (“one-step dissolution”); (b) ternary Au–Ag–Ge sample synthesized using “one-step dissolution”; (c) ternary (Au–Ag)–Ge sample synthesized using “two-step dissolution”; and (d) normalized HR-XRD pattern of ternary samples synthesized using a “two-step dissolution”, showing the phases present before and after the dissolution process.

As can be seen in Figure 2, both dissolution methods produced hierarchical np-Au structures after a 2 h immersion in HNO_3_. The one-step dissolution and two-step dissolution approaches resulted in similar large pores, averaging 228 ± 90 nm, due to the selective dissolution of Ge lamellae. This similarity suggests that the removal of Ge, whether by acid or base, does not influence the morphology or the reordering of the Au–Ag atoms in the surrounding ligaments after dissolution. The second level of porosity, characterized by small pores and formed by the selective dealloying of Ag from the Au–Ag lamellae, also appeared in both dissolution approaches. These pores averaged 35.1 ± 23.7 nm for the one-step approach (Figure 2b) and 32.1 ± 19.2 nm for the two-step approach (Figure 2c). Notably, the two-step dissolution approach produced slightly smaller average pore sizes and a noticeably higher porosity of 47.6% compared with the 39.5% porosity in the one-step approach sample (Table S1). These findings indicate that both dissolution approaches can be used to form hierarchical np-Au morphology, yet the average pore size and porosity vary depending on the approach.

To determine the phases of the samples before and after dissolution, we performed HR-XRD. As shown in the diffraction pattern in Figure 2d, a small (111)­Ge diffraction peak can be detected postetching, indicating the presence of residual Ge that was not completely removed from the structure. The successful dealloying of Ag from the Au–Ag lamellae is evidenced by the shifts observed in the (111) and (200) diffraction peaks shared by Au and Ag. Specifically, the (200) diffraction peak shifts from 44.6° preetching to 44.4° postetching, and similarly, the (111) diffraction peak shifts from 38.3° to 38.2°. This behavior is consistent with the expected differences in lattice dimensions between pure Au (4.08 Å) and a 1:1 Au–Ag alloy (4.09 Å).

The XRD study confirms that both Ge and Ag are removed from the crystals during the dissolution process (Figure 2d), and that the np morphology is obtained.

In order to understand the reasons behind the morphological changes resulting from different dissolution methods, we measured the concentration of Ag removed from the crystals during dissolution into the nitric acid solution using ICP-OES measurements. Two samples were immersed in the acidic solution for 2 h: one immediately after thermal annealing and the other after the selective dissolution of Ge in the basic solution. The acidic solutions were then diluted to 3% HNO_3_ by using deionized water. For the one-step dissolution sample, the concentration of Ag in the solution was measured at 14.3 mg L^–1^, whereas only 11.6 mg L^–1^ of Ag was detected in the two-step dissolution solution. These results confirm that the one-step dissolution approach leads to a higher dissolution rate of Ag compared to that of two-step dissolution. A possible reason for this difference is discussed below.

Since the concentration of HNO_3_, the size distribution of the crystals, and the Ag concentration in the alloy were identical in both cases, no change in the diffusion of Ag out of the crystals is expected.^19^ The two dissolution methods produced distinct morphologies, indicating that the initial selective Ge dissolution step in the two-step dissolution approach plays a key role in the morphological differences between the two approaches. To verify this hypothesis, we compared samples synthesized by the one-step dissolution and two-step dissolution over different dissolution durations in the HNO_3_ solution. Given that the one-step dissolution produced larger pores and ligaments compared to those of the two-step dissolution, we also examined one-step dissolution samples with shorter immersion times to achieve a finer np morphology. Additionally, we tested two-step dissolution samples with both longer and shorter dissolution durations in HNO_3_, aiming to achieve both coarser and finer porous structures.

The outcome of the one-step dissolution approach was examined as a function of varying immersion durations, namely, 30 min, 1 h, and 2 h. As can be seen in Figure 3, shorter dissolution durations of 30 min and 1 h (Figure 3a,d) resulted in finer np morphology. As expected, a longer dissolution duration led to an increase in the average pore size (Table S1). Moreover, upon examination of the morphology and dissolution propagation within the crystals, we found that the dissolution did not fully penetrate the entire crystal volume. Cross-sectional micrographs obtained using FIB coupled with HR-SEM revealed that in the sample immersed for 30 min (Figure 3d), the dissolution front reached only 1 μm beneath the surface, with both Ge lamellae and nondealloyed Au–Ag ligaments still present inside the crystal. The samples immersed for 1 h presented a similar problem (Figure 3e), with incomplete dissolution, leaving residual Ge and Ag within the crystal. Using cross-sectional images of the samples treated with different dissolution durations, we calculated the dissolution front velocity in the acid solution as 1.03 ± 0.24 nm s^–1^. This estimation suggests that near-complete Ge dissolution from large crystals (approximately 10–15 μm) would require between 1.5 and 2 h, whereas for smaller crystals (<10 μm), 1 h of dissolution should suffice for Ge removal and the formation of relatively smaller pores. Consequently, for crystals smaller than 10 μm, fine hierarchical morphologies can be achieved with the one-step dissolution method using shorter immersion durations in the acid, potentially leading to higher surface area (SA) values (Table S1). However, the large size distribution of droplet-shaped crystals formed during the annealing step presents a significant challenge, as the consistency of the results cannot be guaranteed. Based on these results, we can conclude that while the one-step dissolution approach allow the synthesis of hierarchical np-Au crystals, our ability to tune the morphology and increase the SA is relatively limited, making this approach less preferable.

**Figure 3:**
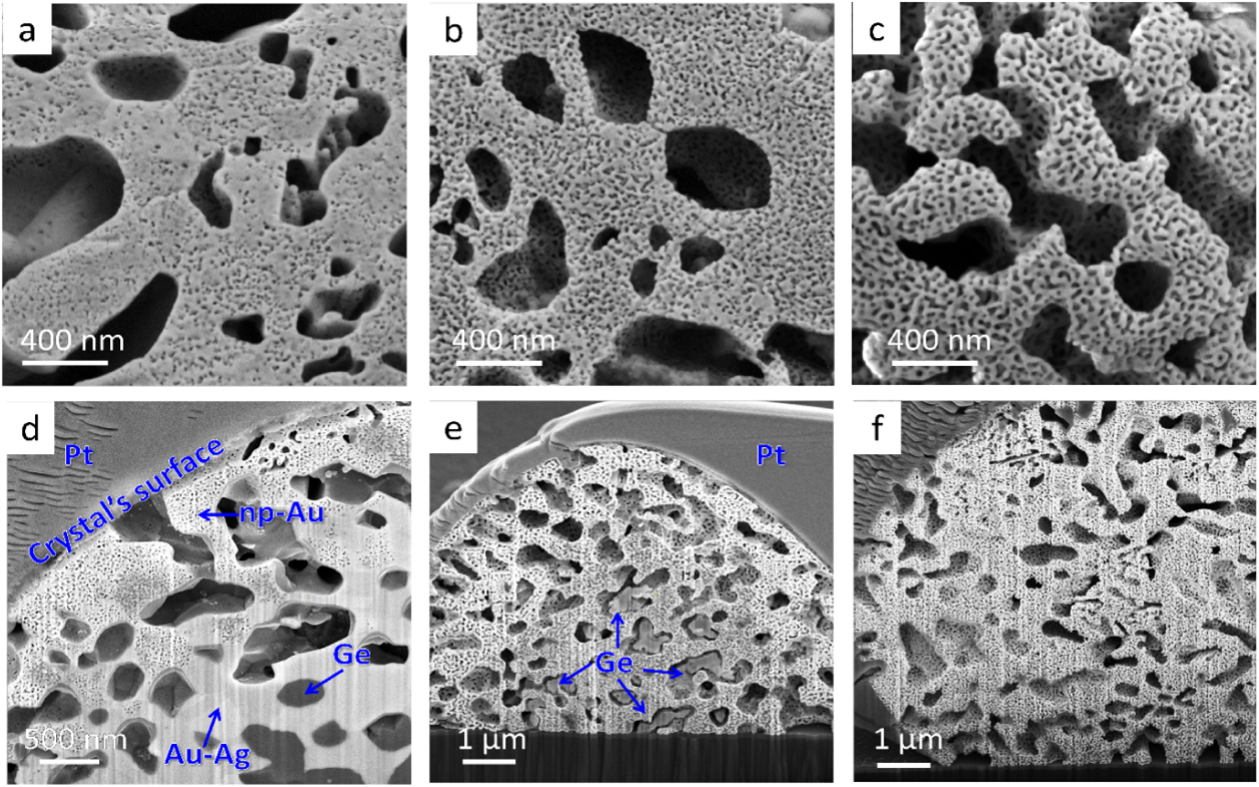
HR-SEM micrographs of (a–c) surface imaging and (d–f) cross sections of hierarchical np-Au crystals synthesized by a one-step dissolution approach for different durations in HNO_3_: (a) and (d) 30 min; (b) and (e) 1 h; (c) and (f) 2 h.

To study the two-step dissolution approach, we synthesized several samples, as presented above. In the first step, all samples were immersed in a basic Piranha solution for 2 h. The duration of the second dissolution stage in HNO_3_ varied among the samples: 1 h, 1:20 h, 1:40 h, 2 h, 3 h, and 5 h. The samples were then imaged using HR-SEM, and as shown in Figure 4, all exhibited hierarchical np morphology. As observed, extending the dissolution time in an acidic solution led to an increase in the average pore size (Table S1). With the average Au–Ag ligament size after the first dissolution step being 228 ± 90 nm and the dissolution front velocity calculated at 1.03 ± 0.24 nm s^–1^, we could conclude that near-complete dissolution of Ag is achievable after 1 h, leaving no residual Ge or Ag within the crystal volume. To confirm this assumption, we examined the cross-section of the sample immersed in HNO_3_ for 1 h. As can be seen in Figure 4h, unlike the one-step dissolution approach, the two-step dissolution approach produces a consistent hierarchical np morphology throughout the entire volume of the crystal after 1 h, confirming that longer immersion durations will ensure near-complete Ag dissolution.

**Figure 4:**
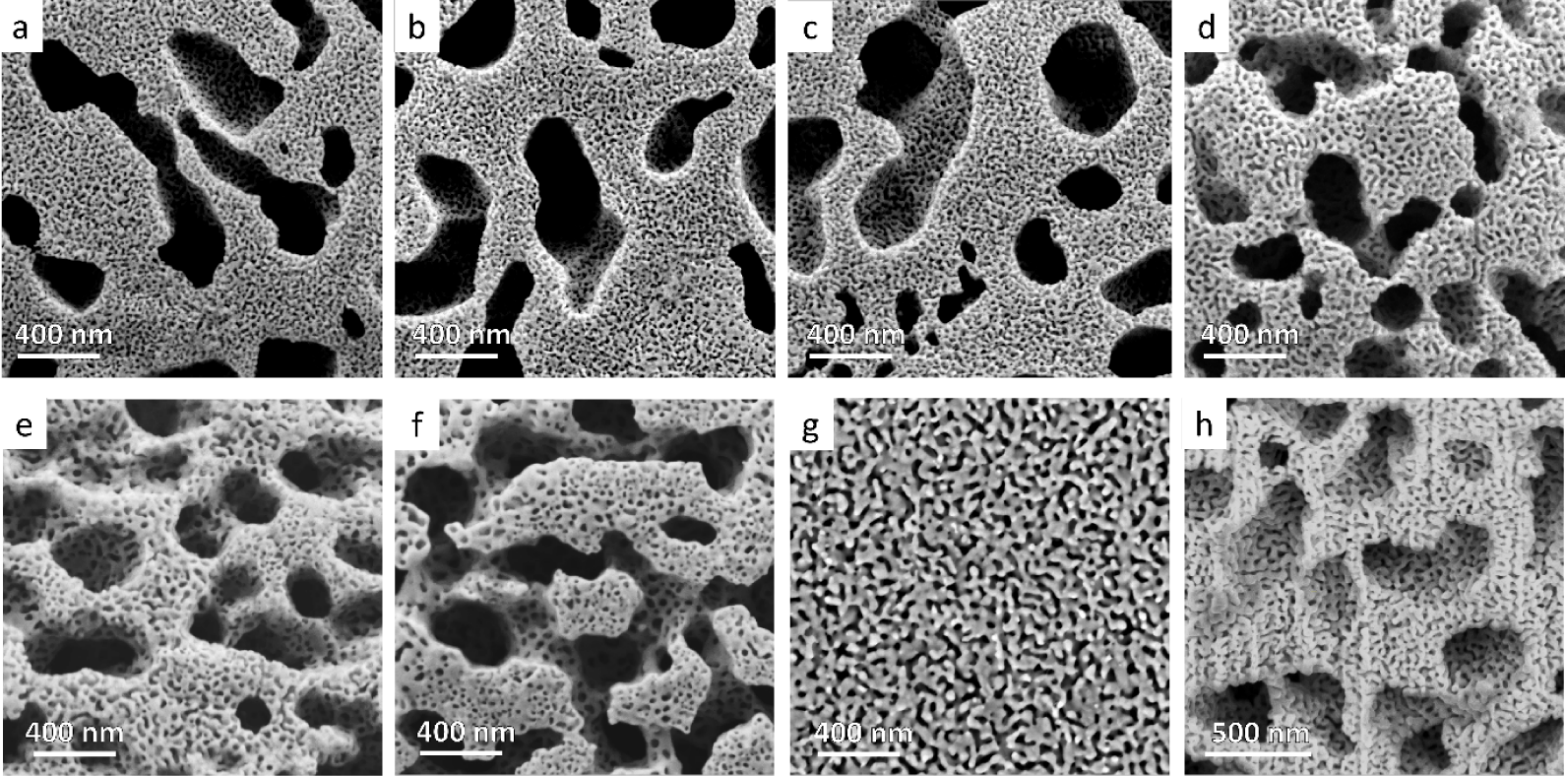
HR-SEM micrographs of surfaces of hierarchical np-Au crystals synthesized by a two-step dissolution approach, immersed for different durations in nitric acid: (a) 1 h; (b) 1:20 h; (c) 1:40 h; (d) 2 h; (e) 3 h; (f) 5 h; (g) conventional np-Au standard sample, synthesized from Au–Ag alloy (without Ge); and (h) cross-sectional imaging of sample (a), confirming the full dissolution of Ge and Ag.

To compare the morphology and pore sizes in our unique hierarchical samples with np-Au crystals synthesized by the conventional approach, we deposited thin Au–Ag layers on a Si/SiO_2_ substrate and thermally annealed them at 850 °C, ensuring dewetting and the formation of Au–Ag droplets on the surface. The sample was then immersed in HNO_3_ for 2 h to develop the np morphology typically obtained via the conventional approach (Figure 4g). A detailed synthesis description is provided in the Experimental Section.

The correlation between dissolution durations and the coarsening of np morphology is a well-known aspect of the dealloying process in many different alloys, including Au–Ag alloys.^44–46^ However, in our case, given that the HNO_3_ concentration was consistent across both dissolution approaches, we did not anticipate a difference in the morphologies observed at the same nitric acid dissolution duration of 2 h (Figures 3 and 4). We propose the following explanation for this discrepancy: in the two-step dissolution process, the initial removal of Ge from the eutectic microstructure results in the formation of large pores and new surfaces of the remaining Au–Ag ligaments. The low-coordination atoms at the newly exposed surfaces, previously serving as the interfaces between the Au–Ag lamellae and the Ge lamellae, may reorder to fill vacancy clusters and reorganize on the surface in atomic steps. The reactive low-coordination Ag atoms on the newly exposed surfaces could potentially react with the H_2_O_2_ and NH_4_OH aqueous basic solution used in the initial step of Ge dissolution, potentially leading to the formation of AgOH and Ag_2_O.^47,48^ The formation of an Ag passivation layer on the exposed surfaces of the crystals could later hinder the full dealloying of Ag from the alloy by blocking the surface of the Au–Ag alloy underneath the passivation layer from the dissolution solution and interfering with the diffusion front propagation of the HNO_3_ solution.^49,50^ This implies that the rate of Au–Ag dealloying in the second step of the two-step dissolution is lower compared to that of the one-step dissolution, which would explain why a finer np morphology is obtained in the two-step process for the same dissolution durations as those in the one-step approach.

To confirm the differing dissolution rates between the two approaches, we performed energy-dispersive X-ray spectroscopy (EDX) measurements on samples synthesized by using both dissolution approaches, each with varying dissolution durations. As can be seen from Table 1, the concentration of residual Ag in the crystals decreases as the immersion time in the dealloying solution increases, as expected. An important finding from these measurements is the similar concentrations of residual Ag in the crystals after 1 h of the one-step approach and after 2 h of the second step of the two-step approach. This result aligns with the very similar morphological features observed for the two samples, as indicated by the average pore size presented in Table S1. Another interesting point is the leveling that occurs in the Ag leaching rate from the crystals after approximately 3 h in HNO_3_ during the two-step dissolution approach. An additional 2 h of dissolution (a total of 5 h) led to only a minor decrease in Ag concentration of 0.8%. This slight variation suggests that the dealloying process has nearly reached its limit. Finally, from the Ag concentration detected in the one-step dissolution sample after 2 h, one can conclude that the dealloying process is almost fully actualized, with the sample reaching a minimal reduction in Ag concentration in the two-step dissolution after 3 h. An additional analysis of the composition in the nanoparticle (NP) cross-section before and after dissolution, as shown in Table 2, yielded similar results, confirming near-complete dissolution throughout the NP structure, including its core.

**Table 1:** Au/Ag (at%/at%) Atomic Ratio after Dissolution for Varying Durations Using 2 Dissolution Approaches

	One-step dissolution			Two-step dissolution		
Duration	30 min	1 h	2 h	2 h	3 h	5 h
**Au**	71.3	89.2	96.4	91.3	97.0	97.8
**Ag**	28.7	10.8	3.6	9.7	3.0	2.2

**Table 2:** Concentrations of Au, Ag, and Ge (Wt%) before Etching and after Dissolution, Using Different Dissolution Approaches

	Before etching (wt %)	One-step dissolution (wt %)	Two-step dissolution (wt %)
**Au**	43.1	99	98.3
**Ag**	43.8	0.8	1.6
**Ge**	13.2	0.2	0.1

It is reasonable to assume that the two-step dissolution method requires shorter dealloying durations, as the first step of Ge dissolution results in the formation of porous crystals with ligaments of an average size of 228 ± 90 nm. As such, the distances required for dealloying in the acidic solution are reduced to the scale of hundreds of nanometers rather than micrometers, as in the case of one-step dissolution. This is further confirmed by comparing the cross-sections of samples immersed in HNO_3_ for similar 1-h durations using the different approaches (Figures 3e and 4h). Given the identical size distribution of crystal droplets for both samples, the presence of large cylindrical pores in the two-step dissolution sample facilitates quicker dealloying, allowing the dealloying front to penetrate the entire volume of the sample more rapidly. However, the formation of passivated AgOH or Ag_2_O layers during the first step of the two-step dissolution method introduces a delay in the second dissolution step. This hindrance at the beginning of the dealloying, along with the reduced effective dealloying duration of the crystals compared to the one-step method, ultimately results in a finer np-Au morphology.

Both the HR-XRD (Figure 2d) and EDX (Table 2) results indicate minimal residual concentrations of Ag and Ge atoms in the hierarchical np-Au crystals. Given that the solubility limit of Ge in Au–Ag exceeds 5%at^41^ and that Au–Ag forms a fully miscible alloy, some residual Ge and Ag atoms are expected within the final lattice of the dealloyed np-Au crystals. Although extending the dissolution duration could further reduce residual elements, prolonged dealloying leads to coarsening of the Au ligaments. Consequently, we opted not to pursue complete removal of the remaining less noble metals, as our goal was to achieve a higher surface area and finer pores and ligaments.

Based on the above results, one can conclude that the preferred method for synthesizing hierarchical np-Au crystals with tunable morphologies, high SA, and a complete 3D bicontinuous structure is the two-step dissolution approach. This approach facilitates the development of a hierarchical nanoparticle (NP) morphology with a finer bicontinuous structure, ensuring near-complete dissolution of Ge and Ag throughout the entire crystal volume.

Another primary objective of this work was to synthesize hierarchical np-Au with fine morphology and high SA to enhance the catalytic activity of the np crystals obtained by the dissolution of eutectic microstructures, surpassing the performance of conventional np-Au samples. It has been previously shown^24,51,52^ that unsupported np-Au crystals with ligaments and pores on a scale of up to 50 nm exhibit high catalytic activity for oxidation reactions. It was also established that the catalytic activity of such a 3D np-Au with higher catalytic activity is attributed to the large number of low-coordination atoms, kinks, and atomic steps present on the curved ligament surfaces. To examine the influence of the hierarchical np structure on catalytic properties, we conducted catalytic experiments involving the decomposition of H_2_O_2_. This reaction was selected as it can be catalyzed by porous metallic catalysts at room temperature, and both the reactants and the products are nontoxic and safe to handle. The chemical reaction of H_2_O_2_ decomposition is as follows:

1 $$2\cdot H_{2}O_{2\left(l\right)}\overset{\mathrm{np}-\mathrm{Au}}{\to }2\cdot H_{2}O_{\left(l\right)}+O_{2\left(g\right)}$$

The catalytic measurements were conducted as follows: a beaker containing a precisely measured amount of H_2_O_2_ solution was weighed both before and after the addition of the np-Au catalyst. To monitor the weight loss of the H_2_O_2_ solution and the release of oxygen, weight measurements were taken hourly. The final changes in weight loss (Δm­(*t*)) were then normalized relative to the amount of catalyst used and its coverage on the Si/SiO_2_ substrate:

2 $$\frac{w_{\mathrm{original}}-w\left(t\right)}{w_{\mathrm{catalyst}}\cdot \left(\mathrm{\% coverage}\right)}=(t)\Delta m$$

where *w*_catalyst_ is the catalyst weight, (% coverage) is the catalyst coverage on the surface of the substrate, *w*_original_ is the weight of the beaker with the solution and catalyst at time *t* = 0, *w*(*t*) is the full weight of the solution, beaker, and catalyst at a specific time, *t*. All samples listed in Table S1 were tested as catalysts for this reaction (the results of the catalytic activity obtained by both dissolution approaches for all samples are provided in Figure S1). A total of four reference samples were also measured for comparison: first, a binary Au–Ge sample after Ge dissolution using HNO_3_ (Figure 2a), representing the catalytic activity of a single level of porosity; second, a standard np-Au sample synthesized by the conventional Au–Ag dealloying (Figure 4g); third, a Si/SiO_2_ substrate to assess the contribution of the substrate to the decomposition reaction without any Au catalyst; and finally, a beaker without a catalyst, serving as a baseline for the natural decomposition of H_2_O_2_ at room temperature under air exposure.

An analysis of the catalytic activity of the samples produced through different synthesis methods (Figure 5a,b) provided several immediate insights. First, the sample synthesized using the binary Au–Ge alloy, having a single level of porosity, displays reduced catalytic activity compared to all hierarchical np-Au samples produced using the ternary Au–Ag–Ge system. Its catalytic activity is one to two orders of magnitude lower than that of the hierarchical samples. The second observation is that the catalytic activity of samples synthesized by the one-step dissolution approach is generally inferior to that of samples produced by the two-step dissolution approach. Finally, the catalytic activity of the conventional np-Au standard sample falls precisely between the levels observed for the two different dissolution approaches, as seen in Figure 5a–c. To further highlight the catalytic activity results and their correlation with SA, we normalized the measured catalytic activity values using conventional np-Au as the reference. This was done by dividing the Δm­(*t*) values from eq 2 for each catalyst by the corresponding value of the conventional np-Au sample, which serves as the standard for H_2_O_2_ decomposition rate:

3 $$\frac{\Delta m{\left(t\right)}_{\mathrm{cat}}}{\Delta m{\left(t\right)}_{\mathrm{con.}}}=\frac{V_{\mathrm{cat}}}{V_{\mathrm{con.}}}$$

where *V*_cat_ is the normalized weight measured at time *t* for a specific catalyst, and *V*_con._ is the normalized weight measured at time *t* for the conventional np-Au sample. As can be seen in Figure 5c, all samples with *V*_cat_/*V*_con._>1 exhibit enhanced catalytic activity for the H_2_O_2_ decomposition compared to the conventional np-Au standard. This observation indicates that all hierarchical np-Au samples synthesized using the two-step dissolution approach of eutectic microstructures have improved catalytic activity over conventional np-Au. The only exception is the sample immersed in HNO_3_ for 5 h, which underwent prolonged coarsening and coalescence, leading to an increased average pore size and decreased porosity, ultimately reducing the sample’s SA. In contrast, for the one-step dissolution approach samples, normalization resulted in *V*_cat_/*V*_con_<1, as expected, since they all exhibited lower SA values compared to the conventional np-Au standard.

**Figure 5:**
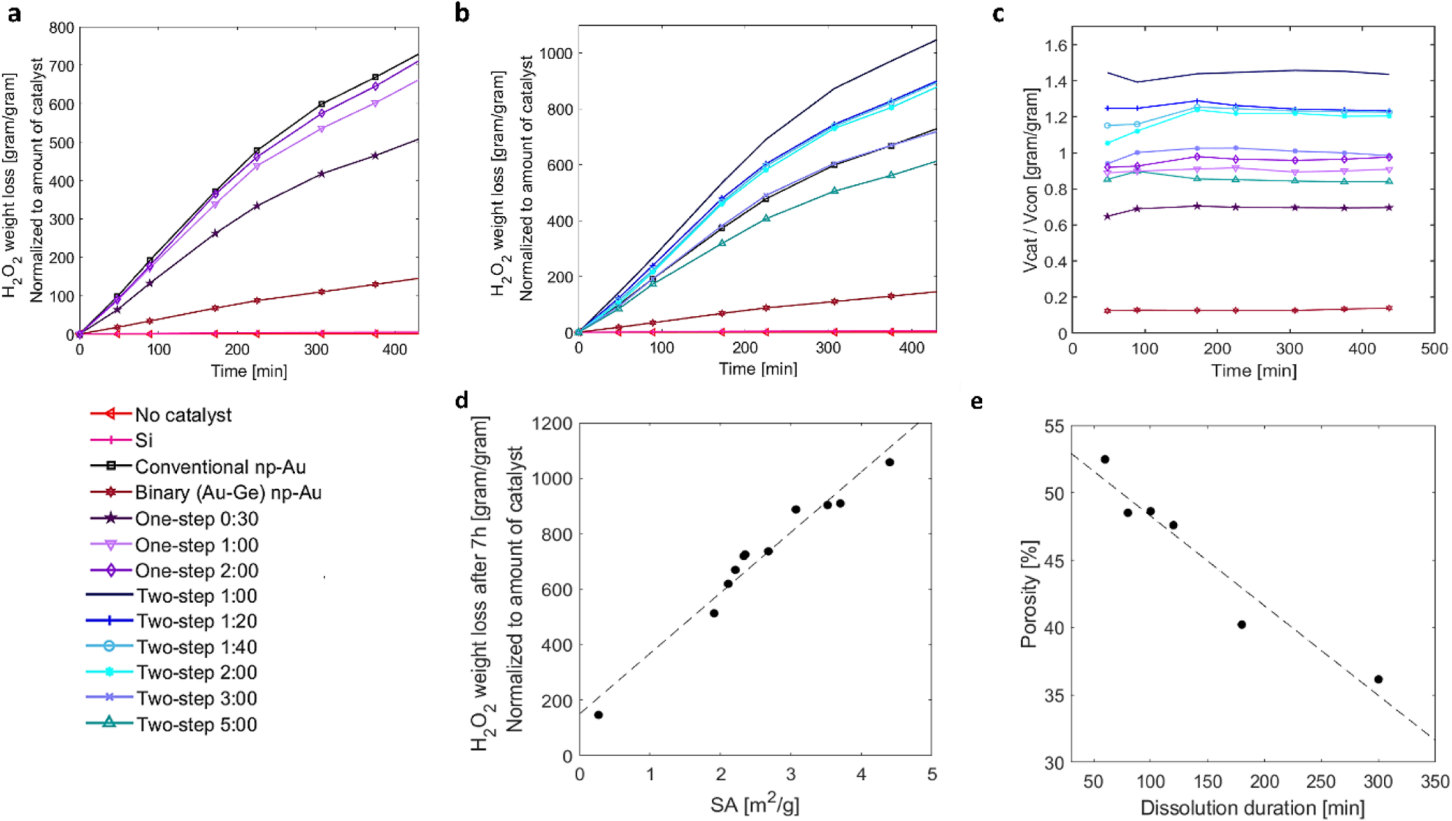
Catalytic measurements for the decomposition reaction of H_2_O_2_ using hierarchical np-Au crystals as catalysts. Results are normalized to the weight and percentage coverage of the catalyst on the substrate. (a) One-step dissolution approach samples; (b) two-step dissolution approach samples; (c) catalytic activity of np-Au catalysts, normalized by the catalytic activity of conventional np-Au; (d) correlation between the SA and the normalized catalytic activity for the H_2_O_2_ decomposition reaction (measured after 7 h); and (e) the average porosity of samples synthesized by two-step dissolution vs the dissolution duration.

The measured catalytic activity of samples synthesized by the one-step approach (Figure 5a), along with their calculated SA values (Table S1), brings into question the expected correlation between SA and catalytic performance. Based on the calculated SA values, the samples immersed in HNO_3_ for 30 min using the one-step method should have shown the highest catalytic activity among the one-step approach samples. Yet, the results indicate that the standard np-Au provided the best decomposition performance, followed by the samples dealloyed for 2 h, 1 h, and finally 30 min. This observation can be explained by the fact that the samples with 1 h and 30 min of dissolution did not undergo full dealloying. Consequently, the SA values calculated according to eq S1 and presented in Table S1 are likely overestimated, meaning the actual SA is significantly lower. The remaining samples confirm the proportional relationship between SA and the catalytic activity results. The conventional np-Au, with an average SA of 3.16 m^2^ g^–1^, outperforms the one-step dissolution sample after 2 h, which has an SA of only 2.33 m^2^ g^–1^.

A similar pattern is observed when the catalytic activity of samples synthesized using the two-step dissolution approach is examined (Figure 5b). The sample with the highest SA of 4.40 m^2^ g^–1^ (1 h of dissolution) presented the best catalytic activity. This was followed by the samples immersed in HNO_3_ for 1:20 and 1:40 h, which exhibited SA values of 3.70 m^2^ g^–1^ and 3.52 m^2^ g^–1^, respectively. When comparing the conventional np-Au to the hierarchical structure produced by the two-step dissolution method, particularly the sample immersed in HNO_3_ for 2 h, it is evident that the hierarchical np-Au crystals are more effective catalysts for this reaction. Despite similar dissolution durations, the hierarchical np-Au exhibited an SA approximately 0.5 m^2^ g^–1^ higher than that of the conventional np-Au. However, extending the dissolution time in the nitric acid to 3 and 5 h results in an increase in average pore size, a decrease in porosity, and a reduction in SA, ultimately leading to diminished catalytic activity.

A notable finding from the catalytic experiments was the significant difference in catalytic activity between hierarchical np-Au crystals synthesized by using different dissolution approaches, even with similar dissolution durations in nitric acid. For example, the sample synthesized using the two-step approach, with 2 h of dissolution in nitric acid, exhibited catalytic activity twice that of the one-step dissolution sample with similar 2 h of dissolution duration. Although the two-step sample had a slightly smaller average pore size, this minor difference alone cannot account for the significant variation in catalytic performance. As discussed above and presented in Table 1, after 2 h in HNO_3_, the one-step dissolution sample had almost 3 times less Ag residue than the two-step dissolution sample, which indicates a longer effective dissolution time. On the other hand, the one-step sample exhibited a significantly lower porosity of 39.5% compared to the two-step approach, which had a porosity of 47.6%. It is known that extended dissolution time, as in the case of the one-step approach, leads to pore coalescence, particularly in the near-surface regions of the crystals, due to the dissolution of thin ligament bridges.^31^ This process causes material reordering and reduces the overall porosity of the sample. A similar decrease in porosity level with increasing dissolution duration was also observed for two-step samples after 1 h, and more notably after 3 and 5 h in nitric acid solution, as shown in Figure 5e and Table S1.

To confirm the correlation between the catalytic activity and the fine-tuned morphological changes that lead to adjustments in SA, we calculated the average SA per unit weight for each sample using the eq S1. The catalytic activity curves (Figure 5a–c) and the average SA values (Table S1) clearly demonstrate a direct relationship: as the SA of the np-Au crystals increases, so does their catalytic activity.

To assess the long-term stability of hierarchical np-Au crystals, catalytic activity experiments were conducted using both newly synthesized and tested samples. Between experiments, the samples were stored under ambient atmospheric conditions without any special storage measures. Prior to each experiment, they were cleaned using acetone, followed by deionized water, and then fully dried before reuse. Across all trials, the results remained highly reproducible, with only minor degradation observed in older samples. Specifically, after 8 months, the catalytic activity of previously used samples decreased by no more than 5%.

These results underscore the considerable advantages of selective dissolution in eutectic microstructures. The np-Au morphology can be precisely tailored by modifying the larger pores and ligaments generated during the dissolution of Ge lamellae, as presented in our previous work.^38^ Additionally, the dimensions of the small pores can be refined through an Au–Ag dissolution step. These combined adjustments enable the formation of np-Au crystals derived from ternary two-phase eutectic microstructures, which exhibit enhanced catalytic activity.

## Conclusions

In this work, we introduce a novel method for creating hierarchical np-Au morphology through the selective dissolution and dealloying of ternary Au–Ag–Ge two-phase eutectic microstructures. Our results demonstrate that employing a two-step dissolution process—initially removing Ge, followed by dealloying the Au–Ag phase through Ag removal—yields a hierarchical NP morphology with a finer bicontinuous structure. This approach results in smaller pores and ligaments, leading to a higher surface area compared to that of conventional NP-Au foam. In contrast, the one-step dissolution process, where Ge and Ag are removed simultaneously, requires a longer effective dissolution time, resulting in pore coarsening and a reduction in SA. The correlation between the dissolution duration and optimal porous morphology is controlled by Ag leaching from the Au–Ag ligaments. In the two-step dissolution approach, the initial Ge removal passivates the remaining Au–Ag lamellae, thereby slowing the second dissolution step and reducing the effective dissolution time in the acidic solution.

However, the formation of large cylindrical-like pores during the initial Ge removal step allows for shorter dealloying durations, ensuring Ag dissolution throughout the crystal depth and resulting in a finer NP-Au morphology. The smaller pores and ligaments of the 3D bicontinuous porous structure, combined with high overall porosity, significantly enhance the surface area (SA), thereby improving the catalytic activity for the decomposition of H_2_O_2_. By precisely controlling morphology through variations in the dissolution duration in nitric acid, we demonstrate a significant enhancement in the catalytic activity of np-Au round crystals derived from the ternary (Au–Ag)-Ge eutectic microstructures. The achieved hierarchical porosity boosts the catalytic performance of novel np-Au by up to 1 order of magnitude compared to binary Au–Ge samples. In addition, we can double the catalytic activity of hierarchical np-Au crystals using different dissolution approaches and durations. Our findings offer a new pathway for synthesizing hierarchical np-Au catalysts via a simple top-down approach using eutectic microstructure dissolution. This technique enables the precise tuning of hierarchical porous morphologies, resulting in considerably enhanced catalytic activity, paving the way for advanced applications in catalysis.

## Supplementary Material



## Notes

Additional details on pore dimension calculations, complete catalysis results, and extended TEM/SEM morphology analysis (PDF)
