# Supplementary material for: Formation of Hierarchical Nanoporous Gold via Selective Dissolution and Dealloying of Ternary (Au–Ag)–Ge Two-Phase Hypereutectic Alloy
Source: Cryst Growth Des. 2025 Jun 11;25(13):4950–60. doi: 10.1021/acs.cgd.5c00382 (PMC12232296; doi:10.1021/acs.cgd.5c00382)
Supplement: Supplementary file 1 [file cg5c00382_si_001.pdf]

Supplementary Information

Formation of Hierarchical Nanoporous Gold *via* Selective Dissolution and Dealloying of Ternary (Au-Ag)-Ge Two-Phase Hypereutectic Alloy

Lotan Portal[a], Iryna Polishchuk[a], Rotem Zilberberg[a], Maria Koifman-Khristosov[a], Alexander Katsman[a], Boaz Pokroy[a,b,c]*

[a] Department of Materials Science and Engineering, Technion – Israel Institute of Technology, 32000 Haifa, Israel.

[b] Russell Berrie Nanotechnology Institute, Technion – Israel Institute of Technology, 32000 Haifa, Israel.

[c] The Nancy and Stephen Grand Technion Energy Program, Technion − Israel Institute of Technology, Haifa 3200003, Israel

* Correspondence to: bpokroy@technion.ac.il

Average pore size and porosity were measured for all samples using the ImageJ program by performing image analysis on HR-SEM images of np-Au samples. An average of 400-500 pores were measured for each to provide the values presented in Table S1. The surface area (SA) values were calculated as follows:

$$\frac{\kappa\theta}{\overline{R}\rho_{Au}}SA = \quad (1S)$$

where $\overline{R_0}$ is the average radius of pores, θ is the porosity, ρ_{Au} is the density of Au, and κ is a geometry-dependent numerical coefficient. For cylindrical-like pores, $\kappa = 2$, while for spherical-like pores $\kappa = 3$. For the sake of simplicity and comparability of results in SA calculations, we used $\kappa = 2$.

Table S1 Summary of average pore size, porosity, and SA for samples synthesized by different dissolution approaches for different durations.

**Dissolution approach**	**Dissolution duration [h]**	**HRSEM micrograph**	$\overline{R_0}$ **[nm]**	θ **[%]**	**SA** $[m^2\ g^{-1}]$
**One-step**	0:30	Figure 3a,d	22.6 ± 20.1	20.8	1.91
	1:00	Figure 3b,e	33.1 ± 21.8	35.3	2.21
	2:00	Figure 3d,f	35.1 ± 23.7	39.5	2.33
**Two-step**	1:00	Figure 4a,h	24.7 ± 14.5	52.5	4.40
	1:20	Figure 4b	27.2 ± 14.9	48.5	3.70
	1:40	Figure 4c	28.6 ± 14.2	48.6	3.52
	2:00	Figure 4d	32.1 ±19.2	47.6	3.07
	3:00	Figure 4e	35.5 ± 19.5	40.2	2.35
	5:00	Figure 4f	35.4 ± 27.7	36.2	2.11
**Conventional np-Au**	2:00	Figure 4g	34.6 ± 7.0	40.9	2.68

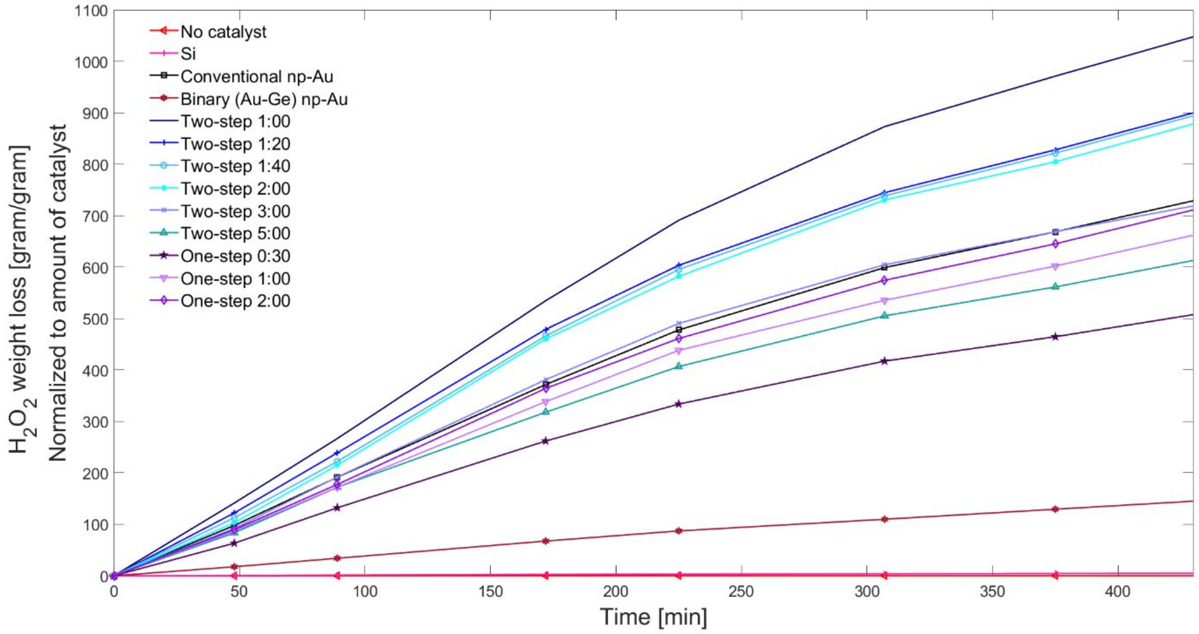

Figure S1 The full catalytic results for the decomposition reaction of H_2O_2 using hierarchical np-Au crystals. The decomposition of H2O2 is normalized to the amount of catalyst used and catalyst coverage on the Si/SiO2 substrate.

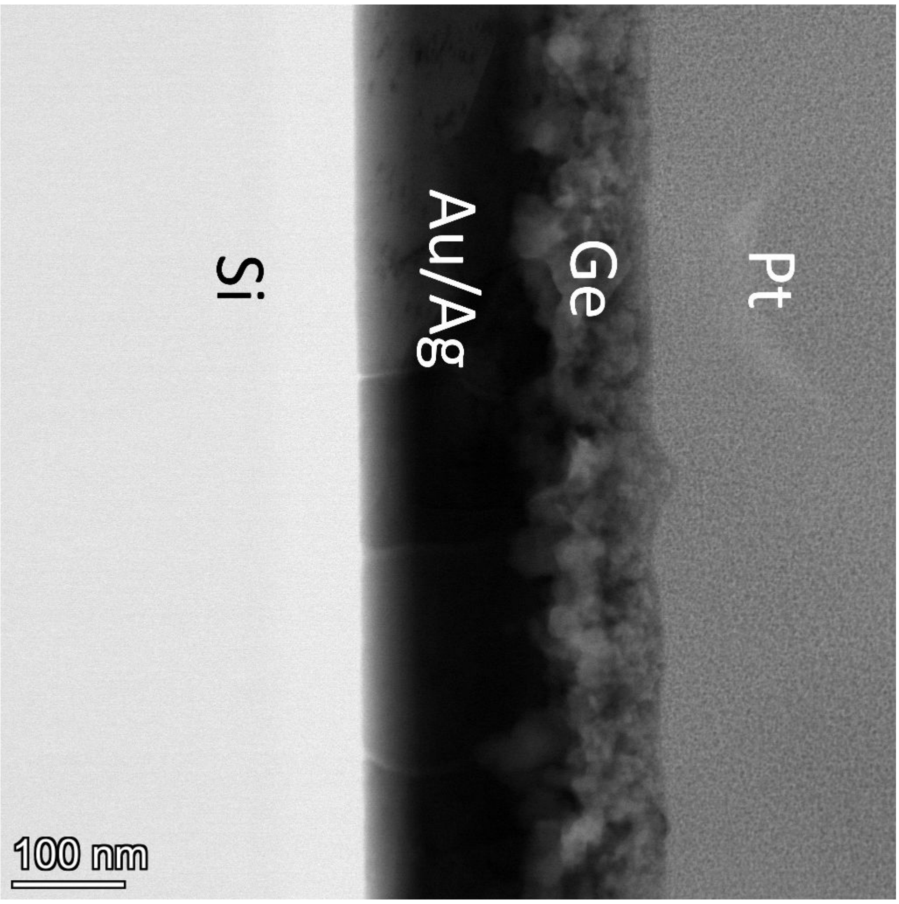

Figure S2 High-resolution transmission electron microscopy (HR-TEM) micrograph of cross-sectional lamella presenting the thin Au/Ag and Ge layers post evaporation on the wafer. Layer thickness was measured at several locations to calculate the final sample compositions.

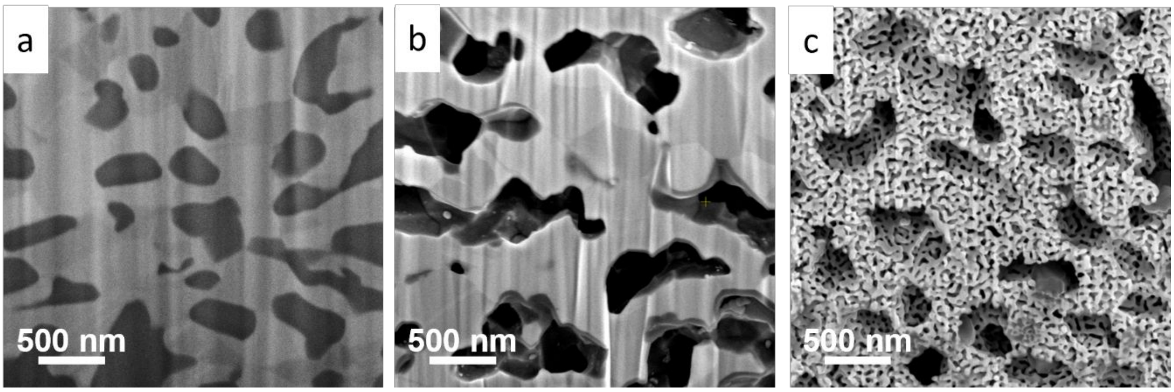

Figure S3 HR-SEM micrographs of cross sections of a droplet-shaped particle formed from the ternary (Au-Ag)-Ge hypereutectic melt (a) before dissolution (b) after Ge dissolution and (c) after a two-step dissolution of both Ge and Ag.
